# Effect of Oral Bisphosphonate Drug Holiday on Mortality Following Hip Fracture

**DOI:** 10.1210/clinem/dgae272

**Published:** 2024-04-17

**Authors:** Miriam T Y Leung, Justin P Turner, Clara Marquina, Jenni Ilomaki, Tim Tran, J Simon Bell

**Affiliations:** Centre for Medicine Use and Safety, Faculty of Pharmacy and Pharmaceutical Sciences, Monash University, Parkville, Melbourne, VIC 3052, Australia; Centre for Medicine Use and Safety, Faculty of Pharmacy and Pharmaceutical Sciences, Monash University, Parkville, Melbourne, VIC 3052, Australia; Faculty of Pharmacy, University of Montreal, Montreal, QC H3C 3J7, Canada; Centre de recherche, Institut Universitaire de gériatrie de Montréal, Montreal, QC H3W 1W5, Canada; Faculty of Pharmacy, Laval University, Quebec City, QC G1V 0A6, Canada; Centre for Medicine Use and Safety, Faculty of Pharmacy and Pharmaceutical Sciences, Monash University, Parkville, Melbourne, VIC 3052, Australia; Centre for Medicine Use and Safety, Faculty of Pharmacy and Pharmaceutical Sciences, Monash University, Parkville, Melbourne, VIC 3052, Australia; Department of Epidemiology and Preventive Medicine, Monash University, Melbourne, VIC 3004, Australia; Pharmacy Department, Austin Health, Heidelberg, Melbourne, VIC 3084, Australia; Centre for Medicine Use and Safety, Faculty of Pharmacy and Pharmaceutical Sciences, Monash University, Parkville, Melbourne, VIC 3052, Australia; Department of Epidemiology and Preventive Medicine, Monash University, Melbourne, VIC 3004, Australia; Faculty of Health Sciences, University of Eastern Finland, Kuopio, FI-70211, Finland

**Keywords:** antiresorptive medication, bisphosphonate, osteoporosis, hip fracture

## Abstract

**Context:**

Current clinical guidelines recommend a drug holiday after extended use of oral bisphosphonates. However, no studies have investigated the effect of drug holidays before hip fractures on postfracture mortality.

**Objective:**

This work aimed to investigate the effect of a drug holiday on postfracture mortality in patients with extended use of oral bisphosphonates.

**Methods:**

This retrospective, population-based cohort study took place among all patients with hip fractures in Victoria, Australia, from 2014 to 2018. Patients were adherent to oral alendronate or risedronate for 5 years or more prior to hip fracture. Group-based trajectory modeling categorized patients into different bisphosphonate usage after 5-year good adherence. The main outcome measure was postfracture mortality.

**Results:**

We identified 365 patients with good adherence (medication possession ratio ≥80%) to oral alendronate/risedronate for 5 years or more. Most patients (69%) continued to use oral bisphosphonates until admission for hip fracture; 17% had discontinued for 1 year and 14% had discontinued for 2 years. Postfracture mortality was higher in patients who had discontinued risedronate for 1 year (hazard ratio [HR] 2.37; 95% CI, 1.24-4.53) and 2 years (HR 3.08; 95% CI, 1.48-6.41) prior to hip fracture. No increase or decrease in postfracture mortality was observed in patients who had discontinued alendronate for 1 year (HR 0.59; 95% CI, 0.29-1.18) or 2 years (HR 1.05; 95% CI, 0.57-1.93) prior to hip fracture.

**Conclusion:**

Postfracture mortality is higher in people who discontinue risedronate, but not alendronate, for 1 or 2 years after being adherent to treatment for at least 5 years. The type of bisphosphonate may be a factor to consider when planning drug holidays.

Osteoporosis is a growing public health concern globally, with hip fractures being associated with the highest costs and mortality among all osteoporotic fractures (1-4). The number of hip fractures is projected to double by 2050 globally (5). In Australia, the absolute number of hip fractures increased by 20% between 2012 and 2017, with a 1-year mortality rate of 25% over this period (6). Bisphosphonates are cost-effective for preventing osteoporotic fractures (7). Emerging evidence suggests that bisphosphonates may also have extraskeletal benefits, including protecting against fracture sequelae such as postfracture mortality (8, 9).

Current clinical guidelines suggest that a drug holiday can be considered in patients at low risk of osteoporotic fractures after 5 years of oral bisphosphonate use (10, 11). This is to minimize the risk of atypical femoral fractures observed with long-term use. Atypical femoral fractures are associated with a 1-year mortality risk of 10% (12). Additionally, long-term use has been associated with an increased risk of osteonecrosis of the jaw, especially in patients using parenteral bisphosphonates (11, 13). Evidence suggests that the preventive effect on osteoporotic fractures may be maintained after discontinuation following 5-year use of oral bisphosphonates (11, 13). However, evidence related to the preferred length of the drug holiday and when to restart bisphosphonates remains weak (10, 11, 13).

The existing evidence on drug holidays for oral bisphosphonates is mainly for alendronate (13). While risedronate users reported earlier significant decrease in bone mineral density (BMD) and more rapid increase in bone turnover rate than alendronate users, no clinical trial data are available beyond 1 year (13). Only 2 observational studies on drug holidays have reported results for individual bisphosphonates. Both studies reported higher risks of fractures during drug holidays in risedronate users than alendronate users (14, 15).

The current evidence on drug holidays mainly relates to changes in BMD or fracture rates rather than fracture sequelae (13). While increase in bone turnover markers or decline in BMD are indicative of bone loss during drug holidays (16, 17), these declines may not reflect fracture rates or sequelae (18). While a prospective cohort study found an insignificant increase of mortality rate in those who discontinued bisphosphonates compared to those who continued, it did not investigate postfracture mortality nor risk of individual bisphosphonate separately (19). Evidence on the possible contribution to postfracture mortality may provide clinicians and patients with new knowledge about risks and benefits when considering treatment continuation or discontinuation after extended oral bisphosphonate use.

The objective of our study was to investigate the effect of different lengths of drug holidays on mortality following hip fractures in patients with good adherence to alendronate or risedronate for at least 5 years prior to the drug holiday.

## Materials and Methods

### Study Design and Data Sources

This was a population-based, retrospective cohort study. Our study population comprised all patients aged 50 years and older admitted to a public or private hospital for hip fracture in Victoria, Australia, from July 2014 to June 2018. Our data set was established from linked population-based databases, including the Victorian Admitted Episodes Dataset (VAED), Victorian Emergency Minimum Dataset (VEMD), Pharmaceutical Benefits Scheme (PBS), and National Death Index (NDI). The VAED contains information on all public and private hospital admissions in Victoria, Australia. Information available includes demographics, diagnoses, location, and type of residences. Patients with hip fracture were identified from the VAED. Records for these patients were linked to records in the VEMD, PBS, and NDI. The VEMD contains information including diagnoses from visits to emergency department with or without hospitalization. The PBS data set captures records of all subsidized medications dispensed at hospital discharge, outpatient clinics, or community pharmacies. The NDI consolidates data from all death registries across Australia. Data linkage was performed by the Australian Institute of Health and Welfare and the Centre for Victorian Data Linkage. The Centre for Victorian Data Linkage is part of the Victorian Agency for Health Information. Data linkage was performed using stepwise deterministic data linkage using personal identifiers including names, addresses, and Medicare number (a unique national public health insurance identifier) (20). Data for our cohort were available from July 1, 2006 to June 30, 2018 for VAED, VEMD, and PBS; while data were available from July 1, 2012 to June 30, 2018 for the NDI. Details of the data sets and data linkage have been described previously (6, 21).

### Study Cohort

The study cohort comprised patients with a principal diagnosis of hip fracture (International Statistical Classification of Diseases and Related Health Problems, Tenth Revision, Australian Modification [ICD-10-AM] S72.0-S72.2) (22-25). We included patients who had at least 8 years of prefracture medication dispensing data for alendronate (World Health Organization Anatomical Therapeutic Chemical Classification [ATC] M05BA04, M05BB03, M05BB05, M05BB06) or risedronate (ATC M05BA07, M05BB02, M05BB04, M05BB07). Patients with diagnoses for hip fractures or cancer (ICD-10-AM C00-C99) within 5 years prior to their index hip fracture were excluded. Patients who switched bisphosphonates or were dispensed other antiosteoporosis medications (ie, raloxifene [ATC G03XC01], hormonal replacement therapy for women [ATC G03C, G03D, G03F, G02BA03], other medications affecting bone structure and mineralization [ATC M05B], eg, denosumab and strontium) within the 8-year prefracture period were also excluded. Only patients with (i) good adherence (≥80% medication possession ratio [MPR]) to oral bisphosphonate therapy in the first 5 years of the 8-year prefracture period, and (ii) more than 1 dispensing of oral bisphosphonates within the last 3 years of the 8-year prefracture period were included to ensure comparability. Patients with more than 1 month of hospitalization within the last 3 years in the 8-year prefracture period were excluded because PBS dispensing data do not include records of medications dispensed to hospital inpatients.

### Initial Adherence (Medication Possession Ratio 8-3 Years Before Fracture)

**Figure 1.**
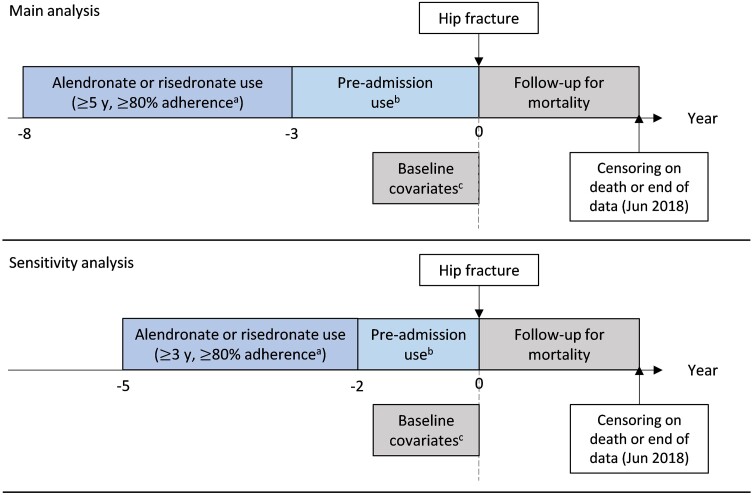
Study diagram for main and sensitivity analyses. ^a^Adherence defined as 80% or greater medication possession ratio over the time period. ^b^Preadmission use categorized by group-based trajectory modeling. ^c^Baseline covariates ascertained at admission for hip fractures; hospital frailty risk scores calculated based on diagnoses within 2 years prior to admission.

Australian guidelines recommend considering a drug holiday after 5 years of continued use (10). Therefore, to identify people who followed the guideline recommendations we identified patients with good adherence to alendronate or risedronate for at least 5 years (8-3 years before fracture). Good adherence was defined as a having high MPR of 80% or greater. The MPR was calculated as the total number of days of supply dispensed divided by the total number of days within the 5-year period. Days of supply were determined from the quantity of medications dispensed based on the dose recommended in the approved prescribing information. The MPR was capped at 100% (Fig. 1).

### Subsequent Use (Trajectories Within 3 Years Before Fracture)

Group-based trajectory modeling was used to categorize bisphosphonate use in the 3 years before fracture following a minimum 5 years of high MPR. Group-based trajectory modeling is an agnostic approach that groups patients with similar dispensing patterns based on their actual dispensings (26). This method accounted for the dynamic nature of bisphosphate dispensing in which patients may discontinue and or reinitiate therapy after different periods of discontinuation (26). Data on oral bisphosphonate dispensing were extracted every 30 days in the 3-year prefracture period. This was because in Australia each dispensing is usually equivalent to 1 month's supply. Group-based trajectory modeling requires medications to have the same dispensing interval, thus medications dispensed less frequently than monthly (eg, denosumab, zoledronic acid) were not included in our model. If more than 1 dispensing was recorded in a 30-day period, the extra dispensing was carried forward to the subsequent month with no dispensing (see Fig. 1). A detailed description of the methods used for group-based trajectories modeling is presented in eMethods and eTables 1 to 6 (27).

### Postfracture Mortality

Dates of death were extracted from the NDI. For patients who died during hospital admission, their date of death was defined from the NDI as the date of death or from the VAED for the date of discharge, whichever occurred first. Time at risk of mortality was defined as time from admission to date of death or end of follow-up, whichever occurred earlier (see Fig. 1).

### Statistical Analysis

Crude survival probabilities were presented with Kaplan-Meier curves, and the log-rank test was used to test for differences among different bisphosphonate use trajectories. Multivariate Cox proportional hazard model was used to estimate the hazard ratio (HR) and 95% CI for mortality among different trajectories of bisphosphonate use following the 5-year period of high MPR. The model was adjusted for factors previously associated with postfracture mortality, including age, sex, frailty, type, and region of residence (6). Frailty was assessed using the validated Hospital Frailty Risk Score (HFRS), a weighted score based on 109 ICD-10-AM diagnosis codes recorded within 2 years prior to admission (28, 29). Patients were categorized as being at no frailty risk (HFRS = 0), low frailty risk (HFRS >0 and < 5), and intermediate to high frailty risk (HFRS ≥5) (28, 29). Data on admission source (eg, residential aged care facility) and geographical region were extracted from records of index hospitalization. The analysis was also adjusted for the fiscal year of the hospitalization to minimize the effect of differences in clinical practice across the years. Analysis stratified by bisphosphonate was conducted to estimate the HRs of each bisphosphonate separately. For overall analysis of all oral bisphosphonates, the interaction term between type of bisphosphonate and the trajectories was added to account for the modifying effect of different bisphosphonates. A sensitivity analysis was conducted to include all available patients using a shortened period for bisphosphonate ascertainment. As available prefracture data were shortened up to 5 years, the first 3 years of the 5-year prefracture period (5-2 years before fracture) was used to identify patients with persistent high MPR and the last 2 years of the 5-year prefracture period (within 2 years before fracture) was used to identify subsequent trajectory use. E-value was calculated to assess residual confounding (30). The study is reported in accordance with the STROBE statement (eTable 7) (27, 31).

### Ethics

The study was approved by the Australian Institute of Health and Welfare Ethics Committee (EO2018-4-468) and Monash University Human Research Ethics Committee (14339).

## Results

Among 18 193 patients hospitalized for primary hip fractures after July 1, 2014, and discharged before June 30, 2018, a total of 1519 patients were dispensed alendronate (n = 900) or risedronate (n = 619) within 8 to 3 years pre fracture and were included in the subsequent medication use assessment (eFig. 1) (27). Among these patients, 27.7% (n = 249) of alendronate users and 24.9% (n = 154) of risedronate users had a high MPR (≥80%). After excluding those who were hospitalized for more than a month and had one or less bisphosphonate dispensing in the 3-year prefracture period, our study included 212 alendronate users and 153 risedronate users. The majority (87.9%, n = 321) were women and more than half (55.9%, n = 204) were aged 85 years or older (Table 1).

**Table 1.** Characteristics of study cohort by oral bisphosphonates^a^

	Overall (n = 365)	Alendronate (n = 212)	Risedronate (n = 153)
Age, n (%), y			
50-64*^a^*	≤5 (≤1.4)	0 (.0)	≤5 (≤3.3)
65-74*^a^*	≤30 (≤8.2)	16 (7.5)	≤15 (≤9.8)
75-84	128 (35.1)	73 (34.4)	55 (35.9)
≥85	204 (55.9)	123 (58.0)	81 (52.9)
Sex, n (%)			
Male	44 (12.1)	21 (9.9)	23 (15.0)
Female	321 (87.9)	191 (90.1)	130 (85.0)
Year			
2014-2015	91 (24.9)	59 (27.8)	32 (20.9)
2015-2016	107 (29.3)	64 (30.2)	43 (28.1)
2016-2017	104 (28.5)	58 (27.4)	46 (30.1)
2017-2018	63 (17.3)	31 (14.6)	32 (20.9)
Admitted from, n (%)			
RACF	30 (8.2)	19 (9.0)	11 (7.2)
Home-dwelling and other*^b^*	335 (91.8)	193 (91.0)	142 (92.8)
Discharged to, n (%)			
RACF	68 (18.6)	48 (22.6)	20 (13.1)
Home-dwelling and other*^b^*	297 (81.4)	164 (77.4)	133 (86.9)
HFRS, n (%)			
0	130 (35.6)	81 (3.2)	49 (32.0)
>0 and <5	146 (40.0)	85 (40.1)	61 (39.9)
5-15*^a^*	83 (22.7)	≤45 (≤21.2)	≤40 (≤32.7)
>15*^a^*	6 (1.6)	≤5 (≤2.4)	≤5 (≤3.3)
Region of residence, n (%)*^c^*			
Metropolitan	265 (72.6)	141 (66.5)	124 (81.0)
Nonmetropolitan	100 (27.4)	71 (33.5)	29 (19.0)

Abbreviations: HFRS, Hospital Frailty Risk Score; RACF, residential aged care facilities.

^*a*^Suppression of small population (ie, conversion of small numbers to number ranges) is required by Australian Institute of Health and Welfare due to privacy considerations.

^*b*^Including discharge to private residences, transition care program, mental health accommodation, and transfers from other health care organizations.

^*c*^Based on Department of Health Human Services Region classification. Areas classified as Eastern metropolitan, Southern metropolitan, and Northwestern metropolitan were categorized as metropolitan, while all other regions were categorized as nonmetropolitan.

**Figure 2.**
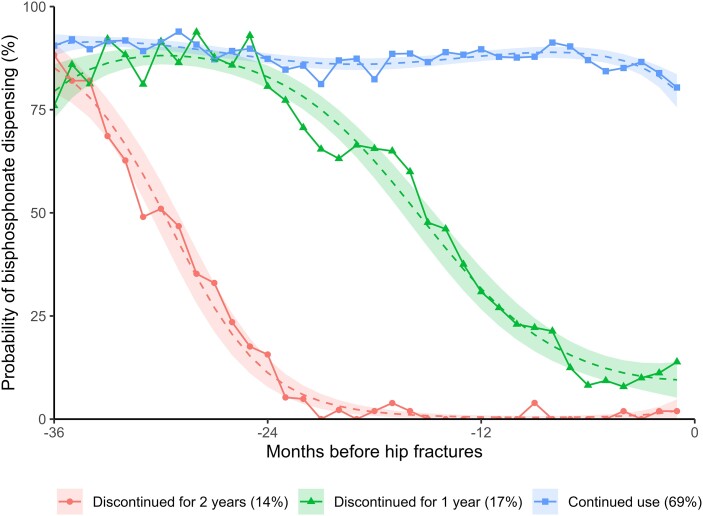
Group-based trajectory model for 3-year prefracture oral bisphosphonate use.

Using group-based trajectory modeling, we identified 3 distinct dispensing patterns in the 3-year prefracture period: (1) continued use (68.8%, n = 251), (2) discontinued for 1 year (17.5%, n = 64), and (3) discontinued for 2 years (13.7%, n = 50) (Fig. 2).

**Figure 3.**
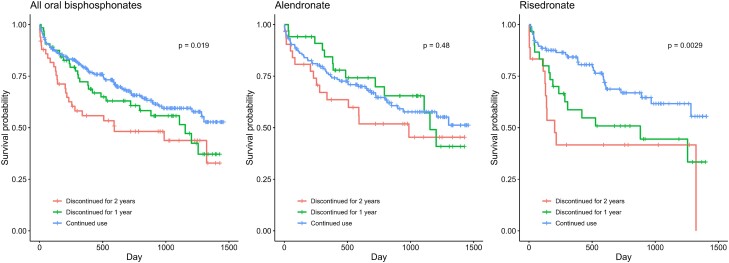
Kaplan-Meier curve of postfracture mortality by individual oral bisphosphonate. p = *P* value of log-rank test.

Over a median follow-up period of 19 months (interquartile range, 7-32 months), 38.2% (n = 81) of alendronate users and 37.9% (n = 58) of risedronate users died after hip fractures. Kaplan-Meier curves illustrate the unadjusted survival rates for patients with different lengths of discontinuation (Fig. 3). After adjustment, no differences in mortality risks were observed in alendronate users who discontinued for 1 year (HR 0.66; 95% CI, 0.35-1.27) or 2 years (HR 1.13; 95% CI, 0.63-2.03). However, significantly higher mortality risks were observed in risedronate users who discontinued for 1 year (HR 2.37; 95% CI, 1.24-4.53) or 2 years (HR 3.08; 95% CI, 1.48-6.41) (Table 2 and eTable 6). The E-value analyses indicated that for risedronate users who discontinued for 1 year and 2 years, only an unmeasured confounder with a minimum association with both discontinuation and mortality of HR 3.73 (95% CI, 1.95) and HR 3.01 (95% CI, 1.59) respectively, would negate the observed increased mortality risks.

**Table 2.** Numbers, mortality rates, absolute rate differences, and adjusted hazard ratios of postfracture mortality for different prefracture bisphosphonate use

	No. of deaths	No. of individuals	Days of follow-up*^a^*, median (IQR)	MR/100 PY (95% CI)	RD/100 PY (95% CI)	HR*^b^* (95% CI)
Overall						
Continued use	84	251	597 (270.5 to 927.5)	19.7 (15.5-23.9)	0	1
Discontinued for 1 y	29	50	533 (240.5 to 1049.25)	26.1 (16.6 to 35.6)	6.41 (−3.99 to 16.82)	0.66 (0.35 to 1.27)
Discontinued for 2 y	26	64	317.5 (128 to 951.25)	36.7 (22.6 to 50.8)	16.98 (2.25 to 31.70)	1.13 (0.63 to 2.03)
Alendronate						
Continued use	53	146	614.5 (267.5 to 973.25)	20.9 (15.2 to 26.5)	0	1
Discontinued for 1 y	13	32	692.5 (384 to 1075.5)	19.4 (8.9 to 30.0)	−1.43 (−13.39 to 10.52)	0.59 (0.29 to 1.18)
Discontinued for 2 y	15	34	526.5 (194 to 978.75)	29.3 (14.5 to 44.1)	8.43 (−7.41 to 24.28)	1.05 (0.57 to 1.93)
Risedronate						
Continued use	31	105	573 (275 to 898)	18.1 (11.7 to 24.4)	0	1
Discontinued for 1 y	16	18	362.5 (172.5 to 861.5)	36.4 (18.6 to 54.2)	18.31 (−0.61 to 37.22)	2.37 (1.24 to 4.53)
Discontinued for 2 y	11	30	172.5 (116.5 to 717)	56.1 (22.9 to 89.2)	38.02 (4.28 to 71.77)	3.08 (1.48 to 6.41)

Abbreviations: HR, hazard ratio; IQR, interquartile range; IR, incidence rate; MR, mortality rate; PY, person-year; RD, rate difference.

^*a*^Days of follow-up are the number of days from admission to date of death or end of study, whichever occurred earlier.

^*b*^Adjusted for year of fracture, sex, age, hospital frailty risk score, type and region of residence, type of oral bisphosphonates, and interaction term between different bisphosphonate use trajectories and type of oral bisphosphonates.

Similar results were observed in sensitivity analysis that included patients with 3 years of high MPR (5-2 years before fracture) for assessing the effect of discontinuation in the 2 years prior to hip fracture. A total of 902 patients (553 alendronate users and 349 risedronate users) were included in the sensitivity analysis (eTable 8, eFig. 2) (27). The 3 distinct dispensing patterns identified in the 2-year prefracture period were (1) continued use (70.0%, n = 631), (2) discontinued for less than 1 year (9.5%, n = 86), and (3) discontinued for 1 year (20.5%, n = 185) (eFig. 3) (27). For discontinuation of less than 1 year, both alendronate users (HR 1.03; 95% CI, 0.77-1.39) and risedronate users (HR 1.18; 95% CI, 0.78-1.78) had similar mortality risk as continued users. However, alendronate users who discontinued for 1 year did not demonstrate higher mortality risk than continued users (HR 1.03; 95% CI, 0.65-1.61), while risedronate users had higher mortality risk (HR 1.66; 95% CI, 1.01-2.73) (eTables 9-10, eFig. 4) (27).

## Discussion

To our knowledge, this is the first study on drug holidays with different oral bisphosphonates and the effect on postfracture mortality. Our study provides new evidence about differential fracture sequelae associated with alendronate and risedronate. We found that after a minimum of 5-year high MPR, risedronate users had a higher risk of postfracture mortality after discontinuation for more than 1 year compared to continued use. In contrast, no increased risk was found in patients using alendronate with up to 2 years of discontinuation. The results remained similar in sensitivity analyses that included patients with at least 3 years of high MPR prior to their drug holiday.

Our results substantiate previous research suggesting that risedronate may have quicker offset than alendronate due to lower bone affinity (32). Studies on bone turnover markers have demonstrated that the benefits of risedronate were no longer apparent after 1 year of discontinuation (18, 33), while other studies have demonstrated sustained benefits of alendronate beyond 3 years of discontinuation (13). Other studies on risks of fractures during discontinuation also demonstrated higher risks in risedronate compared to alendronate (14, 15). A cohort study found a higher risk of fracture in risedronate users when compared to alendronate users after discontinuation for more than 2 years (14). Another cohort study found the risks of osteoporotic fractures were increased both in alendronate and risedronate users who discontinued for more than 2 years, with risedronate users showing higher risk estimates (15). In addition to the effect on risk of fracture, a systematic review also found a greater decrease in postfracture all-cause mortality risk among intravenous bisphosphonate users compared with oral bisphosphonate users when comparing those who persisted on treatment for 3 years to those who ceased (8). This supports the hypothesis that bisphosphonates with higher affinity to bone might have prolonged effects on fracture sequelae beyond risk of fracture.

Our study differs from previous studies in the rate of effect offset (14, 15). We found a higher risk of postfracture mortality in risedronate users after discontinuation of more than just 1 year instead of only after more than 2 years. This difference may be because we investigated fracture sequelae rather than fracture rate or bone turnover markers. It is possible that the outcome of the postfracture mortality rate is more sensitive to drug holidays than other outcomes (14, 15). Additionally, unlike previous observational studies including patients with a minimum of 3-year high MPR use before discontinuation, we did not find an increased risk in alendronate users during our follow-up period (14, 15). It may suggest that alendronate, after the guideline-recommended 5-year high MPR, has a longer sustained effect after discontinuation as demonstrated in randomized controlled trials (13). Future research on more patients with longer follow-up time may be warranted to determine the effect of discontinuing alendronate on postfracture mortality.

It is possible that the different mortality risks found in alendronate users and risedronate users may be due to differential prescribing of alendronate and risedronate. However, recommendations in Australian clinical practice guidelines are the same for alendronate and risedronate (10, 34). Similarly, both alendronate and risedronate have similar treatment indications approved for reimbursement (35, 36). This minimizes the likelihood that patients were prescribed different oral bisphosphonates because of different baseline characteristics. To account for differences in characteristics between alendronate and risedronate users, we adjusted for factors that were associated with 1-year mortality following hip fractures in our previous research (6). We also excluded all patients with cancer who may have had a different indication for using bisphosphonates and mortality risks. We adjusted our analyses for disease burden using the validated HFRS, which is a composite score based on 109 diagnoses codes. We also assessed the distribution of patients receiving medications that could affect bone health, including glucocorticoids, hormone ablation therapy, thiazide and other diuretics, immunosuppressants, proton pump inhibitors, and H2 antagonists. We did not include these medications in the model for postfracture mortality because the prevalence was low and not significantly different among alendronate or risedronate users.

Postfracture mortality may be an important factor to consider during decision-making for undertaking a drug holiday, in addition to risks of atypical femoral fractures and other osteoporotic fracture risks. The risk of atypical femoral fractures was reported to be lower in chronic risedronate than chronic alendronate users (37), which may be another manifestation of the lower bone affinity of risedronate (32). A recent meta-analysis reported patients with atypical femoral fracture have a 10% 1-year mortality rate (12). This is more than half the 1-year risk we reported in our previous study on patients with hip fractures (6). Evidence in relation to possible effects of alendronate and risedronate on the cardiovascular system is mixed; a recent systematic review suggests risedronate, but not alendronate, may confer protection against atherosclerosis (38). Conversely, a Danish cohort study reported that bisphosphonates (predominantly alendronate) reduced cardiovascular events (39), while other reviews have indicated no robust association between bisphosphonates and cardiovascular health (40-42). Our findings contribute to the understanding of the benefit-risk profile of different oral bisphosphonates to guide decision-making regarding drug holidays.

While our findings suggest drug holidays may be associated with lower postfracture survival, our findings do not replace the need for an individualized approach to treatment decision-making. Other factors associated with fracture risks in patients on an oral bisphosphonate drug holiday include older age, fall risk, use of fall-risk–increasing medications, and frailty (10, 11). The risk of osteonecrosis of the jaw may be particularly relevant for patients using parenteral bisphosphonates and undergoing invasive dental procedures (10, 11).

### Strengths and Limitations

Our study has several notable strengths. Our study was conducted among all patients hospitalized for hip fracture in Victoria, Australia. We included only patients meeting current guideline recommendations (having a high MPR over a 5-year period) and excluded patients dispensed other antiosteoporosis medications or hormonal replacement therapy to minimize possible influence from other medications. We also excluded patients who were hospitalized for more than 1 month (13%) because inpatient dispensing data were not available. We acknowledge these patients may have been sicker than those not excluded. Another strength is that we used an agnostic methodological approach, group-based trajectory modeling, to classify patients according to their prefracture pattern of bisphosphonate use. This approach allowed visualization of longitudinal dispensing patterns and avoided potential bias from using arbitrary cutoffs to dichotomize prefracture dispensing. This approach also considered reinitiation and fluctuation in prefracture bisphosphonate dispensing. We adjusted our analyses for a range of factors that have been associated with postfracture mortality (6). However, the risk of osteoporotic fracture is associated with environmental and other confounders that are often unmeasured in observational studies (43). Additionally, by assessing the mortality risk in alendronate and risedronate users separately, alendronate users functioned as a negative control for risedronate users. The disparate results between alendronate and risedronate users provided additional support that the increased mortality risk observed in risedronate users did not merely reflect medication withdrawal in patients with poorer health status thus higher mortality risk.

Our study also has several limitations. We included only patients with high MPR to bisphosphonates for at least 5 years as recommended in clinical guidelines. It is possible that patients with lower MPR or shorter duration of bisphosphonate use may have had worse outcomes. MPR is a valid and widely used adherence measure. However, the medication possession ratio may have overestimated initial 5-year adherence in patients who had their medications dispensed but did not take them, as it was not possible to determine whether dispensed medications were taken by patients. Low treatment adherence has been associated with higher mortality (44). This means that our findings may not be generalizable to patients with poor adherence. Our study was focused on fracture sequelae in patients who experienced hip fractures. The results may be less observable in other osteoporotic fractures with lower postfracture mortality rates (45). It is possible that clinicians may have selectively continued oral bisphosphonates in patients they perceived to be at higher risk of fracture sequelae. However, this would result in a drug holiday being associated with apparent survival benefits, whereas we identified the opposite association for risedronate. We only assessed up to 3 years of subsequent use for drug holidays based on previous evidence showing that risedronate showed significant offset of effect after 1 to 2 years of discontinuation. Further studies with a longer follow-up time are needed to assess the possible effect of longer drug holidays in alendronate users. We also excluded patients who switched to or from other antiosteoporosis medications, and our results may not be generalizable to patients who switched medications. Patients who were hospitalized for more than 1 month during the prefracture trajectory assessment period were also excluded for accurate estimation of their dispensing pattern. This may lead to underestimation of our results as patients who had extended hospitalization were excluded. While our stringent inclusion and exclusion criteria were a strength, they resulted in a smaller sample size, which may reduce our power to detect smaller differences in mortality risks among alendronate users. Our study did not investigate risk factors for different dispensing trajectories. Drug holidays may reflect the decision of the physician or patient, and further studies are needed to understand the reasons for treatment discontinuation.

## Conclusion

Postfracture mortality is higher in people who discontinue risedronate, but not alendronate, for 1 or 2 years after having good adherence to treatment (MPR ≥80%) for at least 5 years. Our findings contribute to our knowledge of the different effect of drug holidays between alendronate and risedronate users.

## Data Availability

Restrictions apply to the availability of some or all data generated or analyzed during this study to preserve patient confidentiality or because they were used under license. The corresponding author will on request detail the restrictions and any conditions under which access to some data may be provided.

## Abbreviations

ATC: World Health Organization Anatomical Therapeutic Chemical Classification
BMD: bone mineral density
HFRS: Hospital Frailty Risk Score
ICD-10-AM: International Statistical Classification of Diseases and Related Health Problems, Tenth Revision, Australian Modification
MPR: medication possession ratio
NDI: National Death Index
PBS: Pharmaceutical Benefits Scheme
VAED: Victorian Admitted Episodes Dataset
VEMD: Victorian Emergency Minimum Dataset
